# A Role for Dogs in Advancing Cancer Immunotherapy Research

**DOI:** 10.3389/fimmu.2019.02935

**Published:** 2020-01-17

**Authors:** Steven Dow

**Affiliations:** Flint Animal Cancer Center, Department of Clinical Sciences, College of Veterinary Medicine and Biomedical Sciences, Colorado State University, Fort Collins, CO, United States

**Keywords:** canine, immune, cell, cytokine, oncology

## Abstract

While rodent cancer models are essential for early proof-of-concept and mechanistic studies for immune therapies, these models have limitations with regards to predicting the ultimate effectiveness of new immunotherapies in humans. As a unique spontaneous, large animal model of cancer, the value of conducting studies in pet dogs with cancer has been increasingly recognized by the research community. This review will therefore summarize key aspects of the dog cancer immunotherapy model and the role that these studies may play in the overall immunotherapy drug research effort. We will focus on cancer types and settings in which the dog model is most likely to impact clinical immuno-oncology research and drug development. Immunological reagent availability is discussed, along with some unique opportunities and challenges associated with the dog immunotherapy model. Overall it is hoped that this review will increase awareness of the dog cancer immunotherapy model and stimulate additional collaborative studies to benefit both man and man's best friend.

## Introduction

Cancer immunotherapy continues to make remarkable strides in just the few years since the first checkpoint molecule targeted therapeutic antibodies were evaluated in trials and approved by the FDA. Indeed, there is the sense by the author and colleagues in the veterinary immune-oncology community (Personal Communication, 2019) that the field of human immune-oncology is advancing so rapidly that new immunotherapy combinations are being evaluated before there is time to determine whether the combinations are truly effective, as judged by evidence of synergistic or additive antitumor activity in realistic animal models (1, 2). Thus, there is a need for additional animal models with which to evaluate new cancer immunotherapies, particularly novel immunotherapy combinations, including immunotherapy combined with targeted therapies, chemotherapy, and radiation therapy. Current rodent cancer models have certain limitations with regards to predicting the ultimate effectiveness of new immunotherapies in humans (3–5).

Increasingly the NIH and pharmaceutical and biotechnology companies are looking to alternative animal models with which to screen immunotherapeutic drugs. The dog spontaneous cancer model has received considerable attention recently (4, 6–13). Several factors drive interest in the dog model. For example, dogs spontaneously develop cancer that resembles human malignancies in many important respects, including phenotype, biological behavioral, histology, mutational signatures and signaling pathways, and immune responses. Indeed, the value of the dog cancer model was recently recognized by the National Institute of Medicine (9).

Therefore, this review will summarize key aspects of the dog cancer model that make it particularly well-suited to evaluating cancer immunotherapies and drug combinations. This will not however be a comprehensive review of all dog cancer immunotherapy studies, which have been reviewed elsewhere and are beyond the scope of this work (3, 14–20). We will instead focus on areas of investigation in which the dog model currently may be most likely to impact clinical immuno-oncology research and new drug development. Reagent availability is also discussed, along with some challenges faced by the dog immunotherapy model along with strategies to overcome these challenges. The intent of this review is to increase awareness of the dog cancer model and stimulate additional studies, which in many cases may benefit both man and man's best friend.

## The Canine Cancer Model and Relevance to Immunological Studies

The uniquely valuable aspects of the canine cancer model have been well-covered in recent reviews (6, 7, 13). With regards to immunological studies in general, there are several key differences between dogs and rodents. For one, dogs are considered an immunologically outbred species, though there are genetic bottlenecks (i.e., limited genotypic or phenotypic diversity within breeds due to extensive inbreeding) for certain breeds of dogs (13). In fact, the availability of dog breeds can in some cases make it possible to map genetic loci to certain immunological traits, as in the example of susceptibility to lymphoma in dogs (21). For example, it was reported that usage of certain VH genes has been associated with improved survival times in canine B cell lymphoma, a dog model for human non-Hodgkin lymphoma (22). Other cancer traits have also been mapped to specific genetic loci in dogs by taking advantage of dog breed genetics (23–26).

Another relevant aspect of the dog model is that the immune system of dogs in cancer immunotherapy studies is typically already very immunologically experienced, with animals having experienced exposure to multiple immunizations during their early years, and to multiple different viral and bacterial infections prior to development of cancer. These immunological events all shape the immune repertoire of dogs and consequently render the dog much more immunologically experienced than rodents raised in sterile cages and fed sterilized water and chow. Dogs also share the same environment of their human companions, and are therefore exposed to many of the same allergens, food antigens, and environmental chemicals (6, 8). Thus, it is not surprising that dogs may react to an immunotherapeutic drug in a different manner than rodents, and behave in many ways more analogously to humans.

Dogs also develop tumors spontaneously, which means that the immune system has typically had weeks to months to recognize the tumor and mount immune responses prior to the appearance of a tumor large enough to diagnose. This long-term exposure to tumor antigens and secreted factors thus educates and conditions the canine immune system in a way that cannot be recapitulated in rodent implanted or induced tumor models (3, 5, 27). Moreover, the canine immune system is much more broadly “educated” which will shape the development of antitumor immunity.

From the standpoint of dosing immunotherapy drugs (other than vaccines), dogs with their similar body sizes and metabolic pathways also fill a gap not currently addressed by rodent studies. Drugs dosed based on weight or body surface area in dogs are much more likely to predict drug activity and toxicity than drugs dosed in mice frequently treated at much higher drug concentrations than can be tolerated by human patients (8). This feature of studies in dogs would be particularly relevant for dosing small molecule drugs and biologics such as monoclonal antibodies, where volume of distribution is critical for determining activity and toxicity (6, 8, 10, 13). The larger size of dogs and their tumors also makes repeated access to blood and tumor tissue biopsies a possibility, which is often important in immunological studies to assess the progression of immune responses, as for example changes in immune infiltrates in tumor tissues.

## Comparison of Dog and Human Immune Cells and Immune Responses

The canine immune system and immune responses in general are very similar to those of humans, with a few notable differences. In broad terms, numbers and proportions of T cells (CD4 and CD8) and B cells in blood of adult dogs closely resemble those of humans (Schalm's Veterinary Hematology, 7th edition and Clinical Immunology of the Dog and Cat, 2nd edition). Moreover, the ratio of CD4 to CD8 T cells (~2:1) in blood and lymph nodes is similar in dogs and humans. Numbers and percentages of neutrophils and monocytes in blood of both species are also very similar. However, recent reagent development for canine NK cells may improve our ability to quantitate dog NK cell responses (28, 29). Dogs also have circulating gamma-delta T cells, though little is known regarding how their numbers may change in disease states (30). Regulatory T cells (CD4+FoxP3+) in dogs have also been defined, and their numbers shown to be significantly increased in dogs with cancer, in both blood and tumor-draining lymph nodes (31–33).

Circulating concentrations of immunoglobulins in adult dogs are approximately the same as those of adult humans, though much less is known about normal immunoglobulin concentrations in young dogs and when final adult IgG concentrations are attained (Schalm's Veterinary Hematology, 7th edition and Clinical Immunology of the Dog and Cat, 2nd edition). Canine IgG molecules can be classified into four functional subclasses (A–D), similar to the human IgG subclasses, with two subclasses capable of binding Fc receptors and two subclasses being Fc functionally negative (34).

T cells in dogs express many of the same co-stimulatory or co-inhibitory molecules as present in humans, including CD28, PD-1, OX40, TIGIT, TIM-3, and Lag3. Like human T cells, canine T cells constitutively express low levels of MHCII, which can be upregulated following T cell activation. In addition, canine effector T cells upregulate production of granzyme B, in addition to CD25 and MHCII (35). Antigen presenting cell (B cells, DC, and monocyte and macrophages) in dogs also share many co-stimulatory or inhibitory molecules with human APC, including MHCII, CD40, CD80, CD86, PD-L1 expression (36–40). In addition, responses to activation, as for example with TLR ligands, is also similar, with upregulated expression of co-stimulatory molecules, and production of pro-inflammatory cytokines such TNF-a, IL-1b, and IL-6, and anti-inflammatory cytokines including IL-10 and TGF-b. In addition, canine monocytes express the chemokine receptor CCR2 and migrate in response to an MCP-1 gradient (41).

An unusual feature of dog neutrophils, which differs from neutrophils in humans, is their expression of CD4 (Schalm's Veterinary Hematology, 7th edition and Clinical Immunology of the Dog and Cat, 2nd edition). The function of CD4 molecule expression by canine neutrophils is unclear, and CD4 is not expressed by other myeloid lineage cells such as monocytes in dogs. Dogs also appear to have a greater abundance of mast cells than humans, especially in mucosal sites such as the skin and airways, and mast cell tumors are much more common in dogs than in humans. Dogs also develop malignancies of cells of the DC and macrophage lineage (e.g., malignant histiosarcoma) at a much higher rate than in humans (e.g., Langerhans histiocytosis) (42–44).

## Selected Canine Cancer Immunotherapy Studies With High Relevance to Human Immuno-oncology

Dogs will never replace rodent cancer models for cancer immunotherapy drug research and development, since early drug screening and mechanism of action studies can realistically only be done in rodent models. However, there are several tumor models where the dog may offer clear advantages, particularly for assessment of new immunotherapies and their potential efficacy against metastatic disease (3, 9, 45). For example, the dog model may be uniquely valuable to address the following issues with respect to cancer immunotherapy: Can new cancer vaccines control advanced metastatic disease?; Can adoptive CAR T cell or NK cell therapy be both safe and active against solid tumors, and what is the safety profile?; How well do tumor microenvironment modifying agents work when combined with existing immunotherapies such as targeted drugs or checkpoint molecule antibodies?; Can checkpoint targeted therapeutics be effectively combined with other cancer treatment modalities (e.g., radiation therapy, cytotoxic chemotherapy) to control or prevent tumor metastases? These examples are discussed in greater detail below. A summary of key recent dog immunotherapy studies is provided in Table 1.

**Table 1:** Summary of relevant canine cancer immunotherapy trials and results.

**Trial**	**Delivery**	**Tumor type**	**Number enrolled**	**Study primary endpoints**	**Secondary endpoints**	**Outcomes**	**References**
Her2 neu vaccine	Listeria vectored (IV)	Osteosarcoma	18	Time to metastasis	T cell responses	Increase OST vs. historical control	(46)
TERT vaccine	AAV vectored (IM)	B cell lymphoma	14	Time to progression, OST	TERT antibodies	Increase OST vs. historical control	(47)
Vaccine plus surgery	Autologous tumor lysate (SC)	Meningioma	11	Tumor progression	Antibody response	No tumor progression over 6 months	(48)
CD20 CAR T	Transduced autologous T cells	B cell lymphoma	1	Safety	Tumor regression	Safely tolerated, partial tumor response	(49)
NK cell ACT	Intratumoral administration	Osteosarcoma	10	Safety, tumor regression	Tumor infiltrates	Improved DFI, NK localization	(50)
Liposomal clodronate	IV, repeat infusions	Soft tissue sarcoma	13	Safety, macrophage depletion	Tumor regression	Macrophage depletion, no tumor responses	(51)
CCR4 blockade	Antagonist antibody (IV)	Bladder cancer	26	Treg infiltrates	Survival, toxicity	Improved OST, Treg depletion	(52)
IDO inhibitor wth XRT	Oral	Melanoma, soft tissue sarcom	5	Safety, tumor response	Reduction in Tregs	Partial tumor response, immune response	(53)
Allogeneic tumor vaccine	Tumor lysate with adjuvant (SC)	Hemangiosarcoma	28	OST, tumor progression	Antibody response	Increase survival vs. historical control	(54)
Bacterial immunotherapy	Attenuated Salmonella (IV)	Multiple tumor types	41	Tumor regression	Bacterial localization	15% overall response rate; dose dependent toxicity	(55)
Local superantigen immunotherapy	Plasmid DNA, intratumoral	Melanoma	26	Tumor regression, OST	Immune infiltrates	Increased survival vs. historical control; CTL activity	(56)
Liposomal MTP	IV, repeat infusions	Osteosarcoma	98	DFI and OST	Macrophage activation	DFI and OST significantly increased	(57)

### Cancer Vaccines

A key question related to the new generation of cancer vaccines currently under development, which is difficult to fully address in rodent models, is whether they can effectively prevent metastatic disease, or control metastases once they develop. As noted above, dogs develop several highly metastatic cancers closely related to human cancers, including in particular osteosarcoma and melanoma (6, 20, 57). These cancers in dogs therefore offer an opportunity to test new cancer vaccine approaches in immunologically realistic settings. As an example, studies are currently underway in dogs with osteosarcoma to determine whether a newly conditionally approved canine osteosarcoma Listeria-vectored vaccine targeting HER2/neu can effectively prevent tumor metastases, and control the growth of macroscopic metastases (46). This novel vaccine approach has demonstrated remarkable early evidence of activity as adjuvant therapy for dogs with osteosarcoma at high risk for tumor metastases. Another example is a plasmid-DNA based tumor vaccine targeting the TERT antigen, which has been evaluated in dogs with lymphoma in combination with CHOP chemotherapy (47, 58). The vaccine has also demonstrated impressive antitumor activity, as reflected in prolonged disease-free intervals compared to relevant chemotherapy only control animals. A number of other cancer vaccine targets are currently being evaluated in canine immunotherapy studies, including the GD3 antigen in canine melanoma studies (59).

### Adoptive Cellular Therapy

Adoptive cell therapy (ACT) with CAR T cells has transformed the treatment of certain leukemias and several other hematopoietic cancers in humans. However, progress in using CAR T cells to treat solid tumors in humans has been more fraught with difficulty, including serious and occasionally fatal toxicities, as well as less overall anti-tumor activity. The adverse events associated with CAR T cell treatment of solid tumors were unfortunately not predicted by rodent cancer models. Given the strong similarities between canine and human immune responses, dogs with solid tumors offer a unique opportunity to evaluate the safety and potential efficacy of new ACTs such as CAR T cell therapy before initiating human clinical trials. Indeed, ACT with CAR T cells has been evaluated in small scale studies in dogs, including CAR T cell studies with a CD20 targeted CAR T cells in dogs with B cell lymphoma (49). Other opportunities for use of the dog tumor model include evaluation of ACT with CAR T or CAR NK cells specific for other widely expressed tumor antigens in dogs, including HER2/neu, EGFR, and GD2 (60). For example, ACT using activated canine NK cells has shown early promise in conjunction with radiation therapy in a dog osteosarcoma model (50).

### Tumor Microenvironment Modification

**Figure 1:**
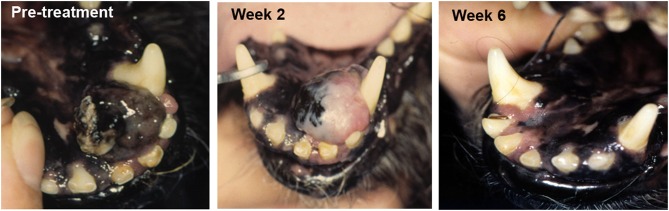
Tumor response to TME modification with a T cell activator. A dog with oral malignant melanoma (left panel) was treated with a series of every 2 week intratumoral injections of plasmid DNA encoding a bacterial superantigen gene (SEB), along with an IL-2 encoding plasmid. Tumor depigmentation was evident after the first injection (middle image) and complete tumor regression was noted after the second intratumoral injection (right panel).

Increasingly studies point to the essential role of the tumor microenvironment (TME) in regulating overall anti-tumor immune responses. Thus, a new wave of therapeutics that target the TME are under development and evaluation in clinical trials. Our studies have identified a wide spectrum of immune responses in tumor tissues of dogs, ranging from highly inflammatory tumors (e.g., melanoma) to tumors that are immunologically “cold” (e.g., soft tissue sarcoma, mast cell tumors, osteosarcoma) (Regan D; Flint Animal Cancer Center, unpublished data). Each of these tumor models in dogs therefore offers the opportunity for evaluation of agents that target the TME, particularly for those designed to remove immune suppressive cells to help activate immunologically “cold” tumors. For example, depleting target tumor-associated macrophages by administration of agents such as liposomal clodronate that deplete tumor macrophages outright has been evaluated in dogs (51, 61). We also found that modifying the TME by direct tumor transfection with a potent T cell activating molecule such as a bacterial superantigen could stimulate T cell infiltration and activation and significant tumor regression in dogs with melanoma [Figure 1; (56)].

A second strategy to eliminate the immune suppressive tumor macrophage population is to prevent their recruitment to tumor tissues by administering agents that block signaling by the chemokine receptor CCR2. Given that there are no currently approved (or affordable) pure CCR2 antagonists available for evaluation in dogs, our group has identified several drugs, most notably angiotensin receptor antagonists (ARBs), that can be repurposed as monocyte migration inhibitors. For example, we reported recently that the ARB losartan exerts potent antitumor activity by blocking signaling via the CCR2 chemokine receptor, thereby inhibiting the recruitment of inflammatory monocytes into tumor tissues, leading to overall tumor macrophage depletion (62). A recently completed clinical trial in dogs with metastatic osteosarcoma treated with high-dose losartan immunotherapy demonstrated significant antitumor activity and systemic suppression of monocyte migration (Regan et al., in review).

As another strategy to modify the TME, it was recently reported that blockade of CCR4 signaling with humanized antibodies could significantly deplete Tregs in a canine model of invasive bladder cancer (52). In that study, treatment with an anti-CCR4 antibody depleted Tregs in bladder tumor tissues in dogs, and was associated with sustained tumor regression, and prolonged survival.

Other groups have investigated indoleamine deoxygenase inhibitors, which target an immune suppressive metabolic pathway in the TME (53, 63). The hypoxic TME in brain cancer can also be modified by administering agents that increase tumor oxygenation in dogs, in conjunction with radiation (64). Thus, the dog cancer model offers multiple opportunities to evaluate TME modulating drugs, particularly because many of these studies have relatively simple PD endpoints and may be of relatively short duration if the primary study endpoints are changes in the TME rather than tumor responses *per se*.

### Checkpoint Molecule Targeted Immunotherapies

Checkpoint targeted therapeutics are far advanced in development and approval for treatment of multiple cancers in humans. As new checkpoint molecule targeted drugs become available in dogs, opportunities exist where the dog model may provide important new information, particularly with respect rational combination therapies of immune targeted drugs given with checkpoint inhibitors. A fully canine PD-1 antibody is currently nearing phase I trial completion in dogs with a variety of different cancers and a product launch is possible in 2020 (40). Other canine checkpoint targeted antibodies are also in the pipeline, including PD-L1 and OX40 antibodies. In addition, several small molecule inhibitors of checkpoint molecules are being investigated in clinical tumor vaccine trials in dogs with brain cancer (64–66).

Thus, the anticipated availability of new checkpoint immunotherapy reagents will make it possible to conduct creative trials in dogs. For example, a number of questions could be addressed, including: Are checkpoint molecule therapeutics effective when administered in an adjuvant setting in dogs with highly metastatic disease such as osteosarcoma or hemangiosarcoma? Or can checkpoint inhibitors be effectively combined with cytotoxic drugs such as CHOP for treatment of lymphoma? Or does co-administration of a checkpoint inhibitor with a tumor vaccine such as the Her2/neu vaccine improve vaccine efficacy in the setting of advanced, bulky tumors? As these examples suggest, a number of important questions related to adjuvant therapy and checkpoint therapy combinations can be addressed in targeted populations of dogs with relevant cancers.

### Other Immunotherapy Approaches

Additional promising cancer immunotherapy strategies are also under evaluation in the dog cancer model. These include the use of viral vectored cytokine delivery approaches (brain cancer), systemic administration of IL-12 nanoparticles (soft tissue sarcoma), bacterial delivered therapeutics (e.g., engineered hypoxia targeting *Salmonella* in soft tissue sarcoma), regulatory T cell depletion with metronomically delivered chemotherapeutics (e.g., toceranib), adoptive transfer of non-specifically activated T cells and IL-15 activated NK cells (osteosarcoma), along with a variety of different cancer vaccines (50, 55, 67). Thus, the canine oncology field has widely embraced the potential for immunotherapy, and it is likely this trend will continue in the future. Data from rigorously conducted trials of immunotherapy in dogs, paired with immune biomarker correlates (9) will help increase the impact of these studies on the human immuno-oncology.

### Challenges for Immunotherapy Studies in Dogs

While there is great promise for studies in dogs with cancer to contribute to the advancement of immunotherapy for both dogs and humans, there are still challenges inherent to the dog immunotherapy model that must be addressed. Among these challenges is a perceived lack of necessary immunological reagents. Though this issue is often cited as a major impediment to immunotherapy studies in dogs, the reality is different (see Tables 2 and 3). For example, there are currently more than sufficient reagents available for evaluating immune responses to cancer, including T and B cell responses (activation, exhaustion, proliferation), monocyte and macrophage responses (numbers, functional phenotype), regulatory T cells (numbers), neutrophils (numbers, function), and NK cells (numbers, function) (Table 2). In addition, there are now a large variety of cytokine reagents for dog studies, including cytokine ELISAs, cytokine multiplexing kits, and antibodies for intracellular cytokine staining and analysis by flow cytometry (Table 3). It is also possible to assess immune responses in archived tissues and cells, using qRT-PCR and Nanostring technology, as well as next generation sequencing technologies (e.g., RNA sequencing).

**Table 2:** Immunological reagents for cell identification and functional assessment in dogs with cancer.

**Molecule**	**Cellular expression**	**Usage**
CD3	T cells	FC, IHC
CD5	T cells	FC
CD4	Th subset, neutrophils	FC, IHC
CD8	Tc subset	FC, IHC
CD9	Myeloid cells, T cells	FC
CD11a	Leukocytes, memory T cells	FC
CD11b	Myeloid cells	FC, IHC
CD11c	DC, some macrophages	FC, IHC
CD14	Monocytes, some neutrophils	FC
CD18	Myeloid cells, MH	FC, IHC
CD19, CD20, CD21	B cells, lymphoma	FC
CD25	Activated T cells, Tregs	FC
CD31	Endothelial cells	IHC
CD34	Hematopoietic stem cells	FC
CD40	APC	FC
CD45	All hematopoietic cells	FC
CD61	Platelets	FC
CD79a	Pre-B cell	IHC
CD86	APC	FC
MHCII	T cells, APC	FC, IHC
FoxP3	Regulatory T cells	FC, IHC
Granzyme B	T cells	FC, IHC
TNF-a	T cells, APC	FC, IHC
IFN-g	T cells, NK cells	FC, IHC
EOMES	T cell (exhausted; memory)	FC
Tim-3	T cell (exhausted)	FC
PD-1	T cell (exhausted); also recently activated	FC
PD-L1	Monocyte, macrophage, DC	FC, IHC
Ki67	Proliferating cells	FC, IHC

**Table 3:** Cytokine reagents for dogs.

**Cytokine**	**Expression**	**Format**
IL-1b	Monocyte, macrophage	ELISA, multiplex
IL-2	T cells, NK cells, B cells	ELISA, multiplex
IL-4	Th2 T cells	ELISA
IL-6	Macrophage, T cells	ELISA, multiplex
IL-7	Multiple	multiplex
IL-8	Multiple	ELISA, multiplex
IL-10	APC, T cells	ELISA
IL-12	APC	ELISA
IL-15	Monocytes, others	multiplex
IL-18	APC	multiplex
MCP-1	Multiple	ELISA, multiplex
TNF-a	APC, T cells	ELISA, multiplex
GM-CSF	Multiple	multiplex
IFN-g	T cell, NK cell	ELISA, multiplex

Another important challenge of the dog model is related to the costs associated with upscaling drugs and immunological reagents for conducting pre-clinical studies in dogs, given their larger body size vs. mice. Moreover, there are substantial costs in terms of personnel (veterinarians, technicians, laboratory personnel) required to support such studies. However, all of these challenges are surmountable, given sufficient support from funding agencies, including more recently the NIH. The setting of realistic expectations at the outset of studies also helps minimize the impacts of these challenges.

### Summary and Conclusions

The era of effective cancer immunotherapy represents a major change in how cancer is treated, and the dog cancer model undoubtedly has an opportunity to play an important role in advancing this field. The value of the dog cancer model for immunotherapy has been demonstrated previously, with the best example being the essential role played by dogs with osteosarcoma development of the non-specific immunotherapeutic L-MTP (liposomal muramyl tripeptide) as an approved immunotherapy for pediatric osteosarcoma (57, 68). The key to leveraging the dog model to advance such studies will be to identify questions that cannot be answered currently in rodent models, and to move nimbly to propose studies that can be informative within a short time frame (months), since the immunotherapy field moves so rapidly. Procuring adequate drug supplies and reagents for large animal studies is also essential. Finally, broad collaborations will always advance the field more effectively than single institution studies, particularly in situations where essential reagents must be shared or where access to patients with certain tumor types is limited. The best possible outcomes will be studies where the results can be translated promptly to benefit both dogs and humans, with their shared tumor types and strong bonds.

## Author Contributions

The author confirms being the sole contributor of this work and has approved it for publication.

### Conflict of Interest

The author declares that the research was conducted in the absence of any commercial or financial relationships that could be construed as a potential conflict of interest.
